# Epidemiology of pediatric schistosomiasis in hard-to-reach areas and populations: a scoping review

**DOI:** 10.1186/s40249-023-01088-x

**Published:** 2023-04-17

**Authors:** Phyllis Munyiva Isaiah, Marta Sólveig Palmeirim, Peter Steinmann

**Affiliations:** 1 Swiss Tropical and Public Health Institute, Kreuzstrasse 2, 4123 Allschwil, Switzerland; 2 University of Basel, Basel, Switzerland

**Keywords:** Schistosomiasis, Prevalence, Epidemiology, Pre-school aged children, Pediatric, Hard-to-reach, Praziquantel

## Abstract

**Background:**

Schistosomiasis affects over 250 million people worldwide. Despite children and the poor being key risk groups, limited research and control activities target pre-school aged children (PSAC) and hard-to-reach populations. As endemic countries shift the goals of their schistosomiasis programs from morbidity control to disease elimination, there is a need for inclusive planning to cover all affected age groups from all geographical areas and populations to achieve sustainable impact and health equity.

**Methods:**

We conducted searches in MEDLINE, Web of Science, Embase (Ovid), and LILACS per the Preferred Reporting Items for Systematic Reviews and Meta-Analyses—Extension for Scoping Reviews (PRISMA-ScR) guidelines. Quality assessment of identified articles was done using the Joanna Briggs Institute Prevalence Critical Appraisal Tool. Relevant study data were extracted from the articles and entered into Microsoft Excel 2016 for descriptive analysis.

**Results:**

From the 17,179 screened articles, we identified 13 eligible studies on schistosomiasis in PSAC living in hard-to-reach areas and populations. All identified studies were from sub-Saharan Africa. The mean sample size of the retained studies was 572, with a balanced sex distribution among the young children sampled in each study. Ten studies investigated *Schistosoma mansoni*, one investigated *Schistosoma haematobium*, while two covered both *S. mansoni* and *S. haematobium* in the target population. The prevalence of *S. mansoni* among PSAC in the included studies was estimated at 12.9% in Ghana, 80.3–90.5% in Kenya, 35.0% in Madagascar, 9.6–78.0% in Senegal, 11.2–35.4% in Sierra Leone, 44.4–54.9% in Tanzania and 39.3–74.9% in Uganda. Out of the three studies that investigated *S. haematobium*, the presence of the infection was reported in only one study carried out in Nigeria. Schistosome infections reported in nearly all studies included in this review were of light intensity. Only one study conducted in Nigeria documented visible hematuria in 17.7% of the PSAC studied.

**Conclusions:**

The findings document the high prevalence of schistosomiasis among PSAC in hard-to-reach populations and underscore the need to consider this population subgroup when designing the expansion of preventive chemotherapy and schistosomiasis control activities.

**Graphical Abstract:**

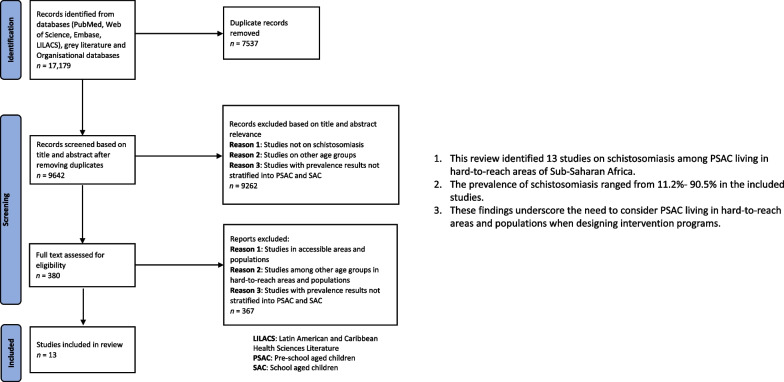

**Supplementary Information:**

The online version contains supplementary material available at 10.1186/s40249-023-01088-x.

## Background

Schistosomiasis is a neglected tropical disease (NTD) that affects over 250 million people globally [1], with children being the most vulnerable group to developing overt disease [2]. Control and monitoring programs for schistosomiasis have been established in almost all endemic countries [3]. However, these programs mainly focus on school-going children, with adults and pre-school aged children (PSAC) largely left untreated [4]. In 2021, the World Health Organization (WHO) estimated that 251 million people required preventive chemotherapy [1] with approximately 10% being children under six years of age [5]. However, there has long been a paucity of data on schistosomiasis among PSAC as, for a long time, the PSAC group was generally categorized as a low-risk group for schistosomiasis [6].

A key challenge for comprehensive schistosomiasis control is reaching all at-risk populations. Main access barriers include geographic, social, and economic conditions. To contribute to the attainment of Sustainable Development Goal 3 and the commitment of global leaders to end neglect and ensure that “no one is left behind” from development progress [7, 8], treatment equity for schistosomiasis must include the most vulnerable groups. These groups include the poorest of the poor, who live in the remotest, hardest-to-reach parts of endemic countries. They often include ethnic minorities, nomadic and migrant populations, and other minority or marginalized populations.

Despite recent epidemiological studies estimating that about 50 million PSAC are infected with schistosomes [9, 10], the true global prevalence and impact of schistosomiasis on the health of PSAC remains largely unquantified [6, 11]. In addition, logistical and operational difficulties in collecting samples from PSAC for diagnosis, the lack of sensitive diagnostics for light schistosomiasis infections, and inadequate data on risk factors in PSAC have further biased schistosomiasis research to focus on school aged children (SAC) and adults [6]. Finally, yet importantly, current donations of the drug of choice for the treatment of schistosomiasis, namely praziquantel, are generally restricted to SAC, and as a consequence, most control programs focus on this group.

In recent years, several studies have documented the occurrence of schistosomiasis and associated morbidity among PSAC [12], encouraging renewed international dialogue, research activities, and policy change by WHO [8]. Current recommendations are to expand schistosomiasis intervention programs to cover all, including PSAC [9]. Although the current formulation of praziquantel is recommended for use among PSAC either on a test-and-treat basis or when delivered through ‘Integrated Management of Childhood Illnesses’ (IMCI) clinics [13], treatment compliance among PSAC is hindered due to the tablets’ large size and bitterness [14].

The Pediatric Praziquantel Consortium, an international public-private partnership that aims to reduce the global disease burden of schistosomiasis by addressing the medical needs of infected PSAC, has developed a pediatric praziquantel formulation. The new formulation tablets are small in size, orally dispersible, and have acceptable palatability [5]. The Consortium is now facilitating its introduction into schistosomiasis control programs in endemic countries through the ADOPT program, an implementation research program that paves the way for the large-scale delivery of the innovative formulation to treat schistosomiasis in all PSAC in endemic countries.

This scoping literature review aimed to document the epidemiology of pediatric schistosomiasis in hard-to-reach areas and populations, providing critical evidence on the need for targeted inclusion of this population when designing the expansion of preventive chemotherapy and schistosomiasis control activities.

## Methods

We defined hard-to-reach areas and populations following Shaghaghi et al. [15], as (i) migrants/island and fishing communities/nomads, (ii) those living in remote physical and geographical locations, and (iii) those living in vulnerable social and economic situations such as minority groups, undocumented persons, socially excluded groups due to language and religious barriers.

This scoping review followed the search and study selection criteria as described in our published protocol [16]. Briefly, the review adopted the proposed five-stage scoping review process of defining the research question, identifying studies, selecting relevant studies, extracting data, and presenting the results as proposed by Arksey et al. [17], while taking into consideration the modifications recommended by Peters et al. [18].

### Eligibility criteria

Only cross-sectional, cohort, and case control studies on schistosomiasis in children under seven years old and living in hard-to-reach areas or belonging to hard-to-reach populations were included. Our searches were not limited in terms of year of publication, geographical region, or language.

### Evidence searches and strategies

We searched four electronic databases, namely MEDLINE, Web of Science, Embase (Ovid), and LILACS for published scientific studies on pediatric schistosomiasis in hard-to-reach areas, using a pre-determined search strategy (Additional file 1). With the guidance of a librarian (University Medical Library—University of Basel), we first developed and optimized a search strategy for PubMed using keywords such as (schistosomiasis OR bilharzia OR katayama fever) AND (children OR preschool child OR infant OR newborn OR under 5 years). This search was then translated using the SR-accelerator tool [19] developed by BOND University to generate the equivalent search terms for Embase (Ovid) and Web of Science. We utilized the (schistosomiasis) AND (children OR preschool child*) search string to search LILACS for published articles. In addition, grey literature and relevant organizational databases were manually searched for relevant articles.

### Study screening and selection criteria

All identified studies were imported into Endnote software X9.3.3 [20], where duplicates were removed. One reviewer (PMI) conducted the first screening to identify studies focusing on schistosomiasis that had been conducted among PSAC based on title and abstract relevance. This was then followed by a full-text review of identified articles, independently conducted by two reviewers (PMI and MSP), to determine whether the study concerned hard-to-reach populations. Any discrepancies in the final identified texts were discussed and where consensus was not reached, advice was sought from the third author (PS).

The review process followed the Preferred Reporting Items for Systematic Reviews and Meta-Analyses—Extension for Scoping Reviews (PRISMA-ScR) guidelines to select the final articles included for review.

### Data extraction and analysis

Extraction of data was done by PMI and reviewed by MSP and PS. The information extracted included: author name(s), year of publication, country of study, sample size, study population age, study population sex, schistosomiasis positive cases, schistosomiasis prevalence, mean eggs per gram or ml (if available), infection intensity classification (if available), schistosome species, type of diagnostic tool, type of hard-to-reach area/population per the definition used in this review, and sampling strategy (Table 1). The extracted information was entered into Microsoft Excel 2016 for descriptive analysis.

**Table 1:** Characteristics of articles included and analysed in this scoping review

	Author name and year	Study country	Study population age	Sample size	Study population sex	Schistosomiasis positive cases	Schistosomiasis prevalence	Mean eggs per gram of stool (epg) or ml urine	Infection intensity classification	Schistosome species	Diagnostic approach	Type of hard-to-reach population/area	Sampling strategy
1	Sassa et al., 2020	Kenya	6–23 months	*n* = 305	Male, *n* = 156	*n* = 287	Kato-Katz, 3.6%	Not specified	Light infections, *n* = 8	*S. mansoni*	Kato-Katz	Island fishing communities	Convenience sampling
					Female, *n* = 149		POC-CCA, 90.5%		Moderate infections, *n* = 1		POC-CCA		Simple random sampling
									Heavy infections, *n* = 1				
2	N'Diaye et al., 2016	Senegal	0–5 years	2008, *n* = 82	Not specified	2008, *n* = 64	2008, 78.0%	Not specified	Not specified	*S. mansoni*	Two direct microscopic examinations	Living in remote physical and geographical locations	Random sampling
				2009, *n* = 61		2009, *n* = 42	2009, 59.0%			*S. haematobium*	Simple microscopy		
				2011, *n* = 38		2011, *n* = 18	2011, 47.4%						
				2013, *n* = 88		2013, *n* = 16	2013, 18.2%						
				2014, *n* = 83		2014, *n* = 8	2014, 9.6%						
				2015, *n* = 108		2015, *n* = 14	2015, 12.9%						
3	Mafiana et al., 2003	Nigeria	< 5 years	*n* = 209	Male, *n* = 123	*n* = 150	71.8%	2.5 eggs/ml urine	Light infections, 62.7%	*S. haematobium*	Sedimentation of urine by gravity	Island fishing communities	All children below 5 years in the study area
					Female, *n* = 86				Heavy infections, 9.6% Visible haematuria, 17.7%				
4	Kabatereine et al., 2004	Uganda	0–4 years	Not specified	Not specified	Not specified	Approximately, 40%	250 epg	Not specified	*S. mansoni*	Kato-Katz	Island fishing communities	Total population
5	Hodges et al., 2012	Sierra Leone	4–5 years	*n* = 421	Male, *n* = 189	Not specified	Overall, 11.2%	Overall, 33.5 epg	Moderate or heavy infections, 8.1%	*S. mansoni*	Kato-Katz	Living in remote physical and geographical locations	Random sampling
					Female, *n* = 232		Highest prevalence, 35.4%						
6	Davis et al., 2015	Kenya	< 7 years	*n* = 201	Male, *n* = 95	*n* = 159	80.3%	Not specified	Light infections, 20.7%	*S. mansoni*	Kato-Katz	Island fishing communities	Convenience sampling
					Female, *n* = 106				Moderate infections, 14.1%		ELISA		
									Heavy infections, 10.1%				
7	Akosah-Brempong et al., 2021	Ghana	< 6 years	*n* = 86	Not specified	*S. mansoni*, *n* = 11	*S. mansoni*, 12.9%	Not specified	Light infections, 89%	*S. mansoni*	Formol-ether concentration technique	Island fishing communities	All eligible children under 15 years
						*S. haematobium*, *n* = 0				*S. haematobium*			
8	Sheehy et al., 2021	Madagascar	2–4 years	*n* = 89	Male, *n* = 45	Kato-Katz, 28/80	Kato-Katz, 35.0%	Kato-Katz, 74.6 epg	Light infections, 78.6%	*S. mansoni*	Kato-Katz	Living in remote physical and geographical locations	Convenience sampling
					Female, *n* = 44	POC-CCA, 56/86	POC-CCA, 67.4%		Moderate infections, 17.9%		Microscopy		
									Heavy infections, 3.6%		Circulating cathodic antigen dipstick		
9	Ruganuza et al., 2015	Tanzania	1–6 years	*n* = 400	Male, *n* = 203	Kato-Katz, 170/383	Kato-Katz, 44.4%	110.6 epg	Moderate infections, 38.2%	*S. mansoni*	Kato-Katz	Island fishing communities	Systematic sampling
					Female, *n* = 197	POC-CCA, 309/386	POC-CCA (trace positive), 80.1%		Heavy infections, 14.7%		POC-CCA		
						POC-CCA, 177/386	POC-CCA (trace negative), 45.9%						
10	Green et al., 2011	Uganda	< 6 years	Lake Albert, *n* = 573	Not specified	Lake Albert, 208/453	Lake Albert, 45.9%	Lake Albert, 230.8 epg	Lake Albert, (> 400 epg, 25/453)	*S. mansoni*	Kato-Katz	Island fishing communities	Not specified
				Lake Victoria, *n* = 455		Lake Victoria, 168/413	Lake Victoria, 40.7%	Lake Victoria, 477.7 epg	Lake Victoria, (> 400 epg, 48/413)				
11	Nalugwa et al., 2017	Uganda	1–5 years	*n* = 916	Male, *n* = 461	*n* = 686	74.9%	294.2 epg	Light infections, *n* = 57.9%	*S. mansoni*	Kato-Katz	Island fishing communities	All eligible PSAC in the study area
					Female, *n* = 455				Moderate infections, *n* = 22.7%				
									Heavy infections, *n* = 19.4%				
12	Nalugwa et al., 2015	Uganda	1–5 years	*n* = 3058	Male, *n* = 1545	*n* = 1203	39.3%	273 epg	Light infections, *n* = 61.0%	*S. mansoni*	Kato-Katz	Island fishing communities	All eligible PSAC in the study area
					Female, *n* = 1513				Moderate infections, *n* = 21.8%				
									Heavy infections, *n* = 17.5%				
13	Mueller et al., 2019	Tanzania	1–5 years	*n* = 71	Male, *n* = 29	*n* = 39	Kato, Katz, 54.9%	91.9 epg	Not specified	*S. mansoni*	Kato-Katz	Island fishing communities	Total population
					Female, *n* = 45		POC-CCA, 95.8%				POC-CCA		

For *S. mansoni*, light infections: 1–99 epg, moderate infections: 100–399 epg, heavy infections: ≥ 400 epg

For *S. haematobium*, light infections: < 50 eggs per 10 ml of urine, heavy infections: ≥ 50 eggs per 10 ml of urine

*POC-CCA* point-of-care circulating cathodic antigen test, *ELISA* enzyme-linked immunosorbent assay, *PSAC* pre-school aged children

### Quality assessment of included literature

The quality of the included articles was assessed using the Joanna Briggs Institute Prevalence Critical Appraisal Tool [21]. All selected studies were scored using the 10 quality control items suggested by the tool. A score of one was awarded for each item fulfilled while a zero score was awarded for each unmet item. Score aggregates were generated and studies were classified as either low (0–3), moderate (4–6), or high (7–10) quality [10] (see Additional file 2).

## Results

**Fig. 1:**
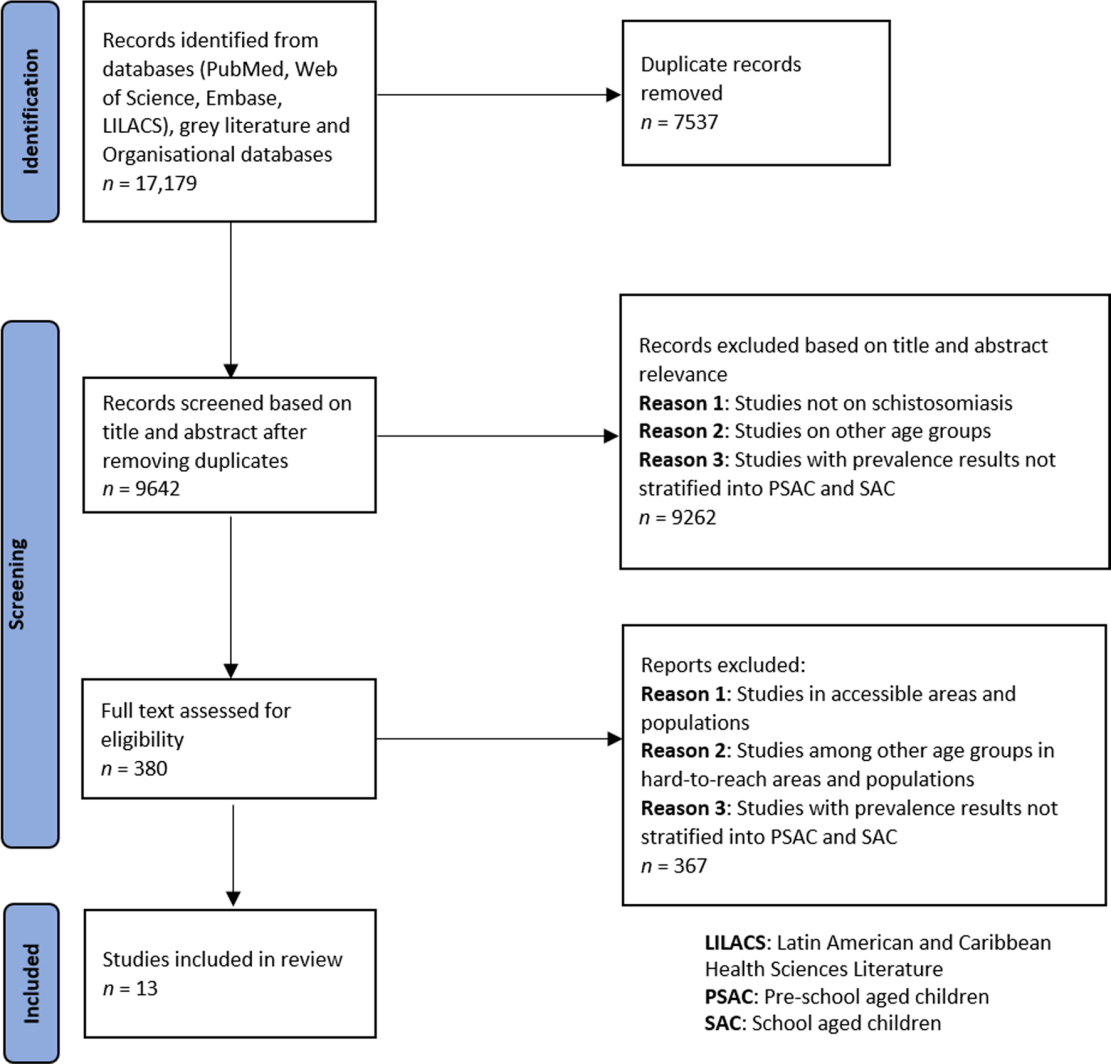
Preferred Reporting Items for Systematic Reviews and Meta-Analyses—Extension for Scoping Reviews flow diagram for a systematic scoping review on the epidemiology of pediatric schistosomiasis in hard-to-reach areas and populations

As shown in Fig. 1 below, we identified 17,179 papers from electronic databases, and after removing duplicates, 9642 papers qualified for screening based on title and abstract relevance. In this step, 9262 papers were excluded while 380 papers were eligible for full-text screening.

Following the full-text screening, we excluded 367 papers due to one or several of the following reasons: (a) studies in accessible areas and populations; (b) studies among other age groups in hard-to-reach areas and populations; (c) studies with prevalence results not stratified into PSAC and SAC. Finally, 13 articles met the inclusion criteria.

### Distribution of included articles based on hard-to-reach area and population definition

All included articles focused on populations living in remote physical and geographical locations, including island fishing communities.

### Sample size and sex distribution

**Fig. 2:**
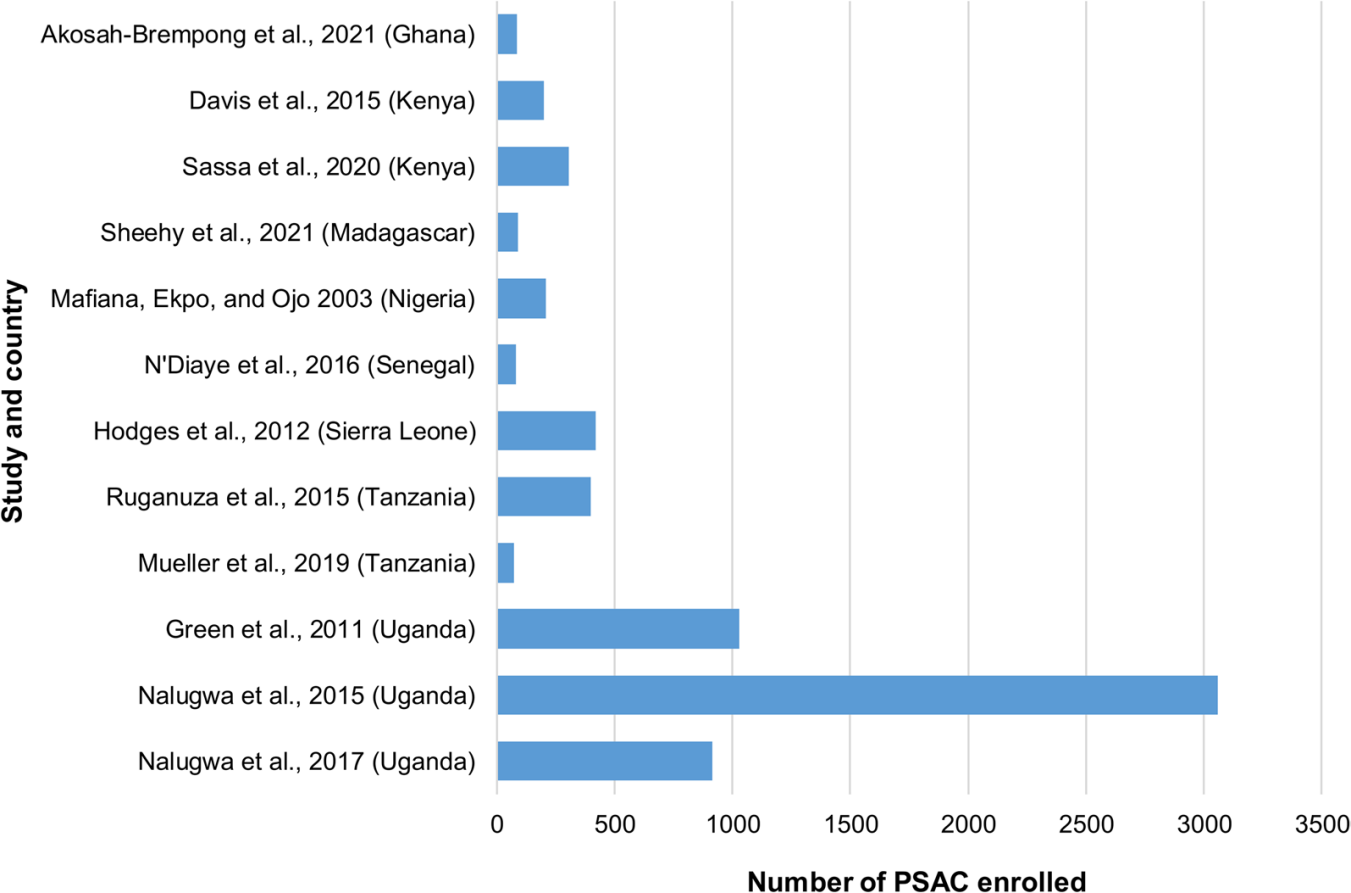
Sample size distribution of all included articles that reported sample size (12 out of 13) per country of study. *PSAC* pre-school aged children

Sample size information was available for 12 out of 13 included articles. Of these, 11 articles reported sample sizes at single time-point surveys, while one [27] reported sample sizes for repeated studies over six years. The mean sample size of reported articles was 572, ranging from 71 to 3058, as shown in Fig. 2. There was a balanced sex distribution in each of the included articles.

### Distribution of included articles by geographic region

All 13 included articles reported data from sub-Saharan Africa. Specifically, the included articles reported data from Ghana (*n* = 1), Kenya (*n* = 2), Madagascar (*n* = 1), Nigeria (*n* = 1), Senegal (*n* = 1), Sierra Leone (*n* = 1), Tanzania (*n* = 2), and Uganda (*n* = 4).

### Study participants’ sampling strategy

Information on the sampling strategy was included in 12 of the 13 selected articles. Notably, the random sampling approach was used for the selection of young children in two studies, while also two studies utilized a convenience sampling method. An additional three studies enrolled all eligible PSAC in the study area, one study sampled children under 15 years and stratified their results into SAC and PSAC, while two studies included total population sampling, including PSAC. One study sampled PSAC systematically, while one used both convenience and random sampling of PSAC.

### Diagnosis of schistosomiasis in included articles

Of the 13 included articles, 10 were conducted in areas where only *Schistosoma mansoni* was endemic while two tested for *S. mansoni* and *Schistosoma haematobium* co-infections in the target population. One study examined *S. haematobium* only. Diagnostic methods were specified in all selected articles. The Kato-Katz method was used to identify *S. mansoni* in a majority of the selected articles (*n* = 10), either alone (*n* = 5), in combination with point-of-care circulating cathodic antigen (POC-CCA; *n* = 4), or in combination with an enzyme-linked immunosorbent assay (ELISA; *n* = 1). Other diagnostic methods used were the formol-ether concentration technique (*n* = 1), and the direct smear microscopic examination (*n* = 1). *S. haematobium* was identified using sedimentation of urine by gravity, urine filtration, and direct microscopy.

### Schistosomiasis prevalence and intensity among PSAC in hard-to-reach areas and populations

All 13 articles reported schistosomiasis prevalence (Table 1). Twelve articles reported only *S. mansoni* infections among the target group while one documented the presence of *S. haematobium*. In one article [22], the Kato-Katz test for *S. mansoni* was complemented by an ELISA test using soluble worm antigen, which did not distinguish between *Schistosoma* species [23]. The prevalence of *S. mansoni* in the included articles was estimated at 12.9% in Ghana [24], 3.6–80.3% in Kenya [22, 25], 35.0% in Madagascar [26], 9.6–78.0% in Senegal [27], 11.2–35.4% in Sierra Leone [28], 44.4–54.9% in Tanzania [29, 30], and 39.3–74.9% in Uganda [12, 31–33].

A study conducted in Nigeria by Mafiana and colleagues [34] documented a prevalence of 71.8% (150/209) among PSAC infected with *S. haematobium.* Two of the included articles, which investigated both *S. mansoni* and *S. haematobium* in Senegal [27] and Ghana [24], did not report any cases of *S. haematobium*, while *S. mansoni* cases were documented in both studies.

We noted discordant prevalence results with POC-CCA (Table 1). For instance, in Kenya, Sassa et al. documented a prevalence of 90.5% with POC-CCA versus 3.6% with Kato-Katz [25], while in Madagascar the *S. mansoni* prevalence was 67.4% with POC-CCA versus 35.0% with Kato-Katz [26]. In Tanzania, Mueller and co-workers established a *S. mansoni* prevalence of 54.9% with Kato-Katz versus 95.8% with POC-CCA [30].

The schistosome infections reported in the 11 studies included in this review were mostly of light intensity. However, in Tanzania, Ruganuza et al. [29] reported that 38.2% and 14.7% of sampled PSAC (*n* = 383) had moderate and heavy infections, respectively. Another study in Uganda [33] reported that out of 3058 PSAC studied, 17.5% had heavy infections.

### Morbidity due to schistosomiasis among PSAC in hard-to-reach areas and populations

In Kenya, Davis et al. [22] documented an association between schistosomiasis and both hepatomegaly and splenomegaly in PSAC. The study also observed persistent hepatomegaly after treatment with praziquantel [22]. In Nigeria, Mafiana and colleagues documented visible hematuria in 17.7% of the PSAC included in the study [34]. Nalugwa et al. evaluated anemia, hepatosplenomegaly, and anthropometric derangements amongst PSAC in Uganda [12]. The study showed that 52.4% of the *S. mansoni* infected children were anemic (mild, moderate, and severe anemia combined) compared to 35.3% of the uninfected children. The study also noted that children infected with *S. mansoni* were more likely to be moderately malnourished than uninfected ones (5.5% versus 1.3%; *P* = 0.007). Liver texture abnormalities were also observed in *S. mansoni-*infected children.

## Discussion

The epidemiology of schistosomiasis among PSAC is poorly researched and documented [35]. Given that many control programs in endemic countries are school-based [36], there is an intervention gap in the PSAC population, which must be addressed if global schistosomiasis control and elimination milestones and the Sustainable Development Goals targets are to be met.

There is increasing recognition that purposeful targeting of hard-to-reach areas and populations is pivotal to the success of disease control and elimination programs. For instance, poliomyelitis elimination programs in India and Nigeria made headway only when specific programs were implemented to vaccinate hard-to-reach groups, including migrant workers and religious minorities [37, 38]. Additionally, previous experiences from malaria programs underscore that equitable access to services and interventions in hard-to-reach areas and populations is critical for malaria elimination and sustaining malaria elimination efforts [39]. To the best of our knowledge, this is the first scoping review that attempts to summarize the available literature on the epidemiology of pediatric schistosomiasis in hard-to-reach areas and populations, aiming to provide evidence for the inclusion of this group in schistosomiasis control and elimination efforts.

In our review, we identified 13 articles published between 2003 and 2021 that focused on schistosomiasis among marginalized, hard-to-reach populations. These studies mainly focused on *S. mansoni*, and were primarily conducted in island fishing communities; there was only limited data from other hard-to-reach areas and populations. Although we identified two studies on nomad populations [40, 41], these were not included in the review, as the authors did not stratify prevalence results into appropriate age groups. Of note, articles from populations living on the shores of the Great Lakes (as opposed to islands) were not included as we felt they did not meet the ‘hard-to-reach’ criterion. Other studies may have not identified their study population as hard-to-reach and were, therefore, considered standard surveys and not included in our review.

We noted fewer studies focusing on *S. haematobium* among PSAC compared to studies investigating *S. mansoni*. This could be due to difficulties in collecting urine samples from PSAC for diagnosis, and previous reports that over the years have mostly documented low intensity infections among PSAC, resulting in fewer studies on *S. haematobium* [42, 43]. A recent study conducted in Zimbabwe [44], demonstrated clinical morbidity markers for *S. haematobium* infection in PSAC, noting that *S. haematobium* affects the respiratory, gastrointestinal, and lymphatic systems, in addition to the genitourinary system. Therefore, we suggest that, as schistosomiasis programs expand, these should include all forms of schistosomiasis among PSAC, and also target those living in hard-to-reach communities.

We observed wide disparities in the published prevalence of schistosomiasis among the target group. In Uganda, two studies conducted amongst fishing communities in Lake Victoria reported prevalences of 39.3% [33] and 74.9% [12] based on the Kato-Katz method, while in Kenya among communities living in the same lake, Sassa et al. [25] found a 3.6% prevalence based on the same diagnostic method. The observed prevalence difference between studies is probably due to heterogeneities in early-life exposure to infected water sources [45, 46].

There is no reason to believe that risk factors are different between different age groups of young children. Infants are likely to already be regularly exposed to infection. In fact, some studies explored the prevalence of *S. mansoni* in children below 2 years of age; in Kenya [25], a very high prevalence (90.5% by POC-CCA) was reported. Similarly, in Senegal, *S. mansoni* infection was already detected in children under 2 years of age [27]. In Nigeria, the prevalence of *S. haematobium* was 71.8% (150/209), with 42.9% of infants infected [34].

Most articles included in the review reported light-intensity *S. mansoni* infections in PSAC based on mean eggs per gram of stool estimates*.* Given that heavy intensity schistosome infections in young children may have long-term developmental consequences [47], there is an urgent need for targeted programs designed to prevent and treat schistosomiasis in this highly vulnerable population. Despite the paucity of data on the morbidity of schistosomiasis among PSAC compared to SAC [6], new studies are shedding light on organ-specific morbidities associated with schistosomiasis and the impact of praziquantel administration [48]. Davis et al. [22] showed that much of the morbidity detected in PSAC was attributed to *S. mansoni* infections, measured by the enlargement of the left liver lobe which is related to infection with *S. mansoni* [49] and is usually associated with acute schistosomiasis [50], especially in children [51], leading to possible risk of wasting and stunting [52].

Critical programmatic and operational barriers affect the deployment of optimal diagnostics for PSAC [6], especially in hard-to-reach areas. The articles included in this review report a predominant use of the Kato-Katz technique for diagnosing intestinal schistosomiasis and urine filtration/sedimentation by gravity for urogenital schistosomiasis, both of which have previously shown low sensitivity in light-intensity infections [23]. These results align with previous studies in Kenya, Tanzania, and Uganda using POC-CCA to identify *S. mansoni* antigen in urine collected from children, which showed higher prevalence rates when compared to the Kato-Katz technique [53, 54]. Another study, which used Kato-Katz and POC-CCA, could only identify the presence of *S. mansoni* infections in children under 10 years old by POC-CCA. [55]. We note that further research is needed to evaluate the accuracy of different diagnostic tools among PSAC for accurate schistosomiasis surveillance and drug efficacy monitoring.

Despite the high prevalence of schistosomiasis in PSAC presented in several of the articles included in this review, there is evidence that it can be reduced when this population is included and closely monitored as part of a comprehensive national schistosomiasis control program. For instance, N’diaye et al. [27] reported a temporal reduction in the prevalence of schistosomiasis in PSAC living in a hard-to-reach population in Senegal, from 78% in 2008 to 12.9% in 2015. To achieve control and elimination of schistosomiasis, WHO suggests that preventive chemotherapy in PSAC is appropriate for those aged ≥ 2 years while for children under 2 years, a test-and-treat approach is recommended. With a pediatric formulation of praziquantel under development, WHO recommendations for PSAC suggest their inclusion in large-scale treatment campaigns in areas where the prevalence is above a 10% threshold [56]. The findings from this review support the recommendation by WHO to systematically consider PSAC in endemic areas, including among hard-to-reach populations when planning schistosomiasis control and elimination interventions. In parallel to chemotherapy, WHO also recommends implementing health education interventions along with improving access to adequate sanitation and safe drinking water as part of an integrated control approach [57].

## Conclusions

This review sheds light on the current state of evidence on the prevalence of schistosomiasis in PSAC in hard-to-reach areas and populations. Our findings are relevant for planning and implementing pediatric schistosomiasis control programs in endemic countries by adapting current intervention protocols. The review also identified data that might aid in improving schistosomiasis programs’ budgeting at national and sub-national levels, and support more accurate praziquantel needs estimation in endemic countries. The expected availability of a new child-friendly praziquantel formulation presents a unique opportunity to accelerate global efforts toward eliminating schistosomiasis.

## Supplementary Information

**Additional file 1.** Databases and search strategies.

**Additional file 2.** Quality assessment report of articles included in the review.

## Data Availability

All data generated or analyzed during this study are included in this published article and its Additional files.

## Abbreviations

ELISA: Enzyme-linked immunosorbent assay
IMCI: Integrated Management of Childhood Illnesses
NTD: Neglected tropical disease
POC-CCA: Point-of-care circulating cathodic antigen
PRISMA-ScR: Preferred Reporting Items for Systematic Reviews and Meta-Analyses—Extension for Scoping Reviews
PSAC: Pre-school aged children
SAC: School aged children
WHO: World Health Organization
