# Supplementary material for: Epidemiology of pediatric schistosomiasis in hard-to-reach areas and populations: a scoping review
Source: Infect Dis Poverty. 2023 Apr 17;12:37. doi: 10.1186/s40249-023-01088-x (PMC10108517; doi:10.1186/s40249-023-01088-x)
Supplement: Supplementary file 1 — Additional file 1. Databases and search strategies. [file 40249_2023_1088_MOESM1_ESM.docx]

**Additional file 1:** Databases and search strategies

| **a.) PubMed Search Query**  (Schistosomiasis[Mesh] OR Schistosom*[tiab] OR Bilharzi*[tiab] OR "blood fluke*"[tiab] OR "snail fever*"[tiab] OR "Katayama fever*"[tiab]) AND (Child[Mesh] OR child[tw] OR children[tw] OR Infant[Mesh] OR infan*[tw] OR newborn*[tw] OR new-born*[tw] OR baby[tw] OR babies[tw] OR suckling*[tw] OR toddler*[tw] OR childhood[tw] OR schoolchild*[tw] OR childcare[tw] OR child-care[tw] OR young[ti] OR youngster*[tw] OR preschool[tw] OR pre-school[tw] OR kid[tw] OR kids[tw] OR boy[tw] OR boys[tw] OR girl*[tw] OR pre-adolescen*[tw] OR schoolage*[tw] OR school-age*[tw] OR schoolboy*[tw] OR schoolgirl*[tw] OR Pediatrics[Mesh] OR Pediatric*[tw] OR Paediatric*[tw] OR (child[all] NOT child[au]) OR children*[all] OR schoolchild*[all] OR "under 5"[tw] OR "<5 year*"[all] OR "<=5 year*"[all] OR infan*[all] OR pediat*[all] OR paediat*[all] OR neonat*[all] OR toddler*[all] OR preteen*[all] OR newborn*[all] OR postneonat*[all] OR postnatal*[all] OR puberty[all] OR preschool*[all] OR suckling*[all] OR juvenile[all] OR "new born*"[all] OR new-born*[all] OR neo-nat*[all] OR neonat*[all] OR perinat*[all] OR underag*[all] OR "under age"[all] OR "under aged"[all] OR youth*[all] OR kinder*[all] OR pubescen*[all] OR prepubescen*[all] OR prepuberty[all] OR "school age"[all] OR "stratified by age"[all] OR schoolage[all] OR "school ages"[all] OR schoolage*[all] OR "one year old"[ti] OR "two year old"[ti] OR "three year old"[ti] OR "four year old"[ti] OR "five year old"[ti] OR "six year old"[ti] OR "1 year old"[ti] OR "2 year old"[ti] OR "3 year old"[ti] OR "4 year old"[ti] OR "5 year old"[ti] OR "6 year old"[ti] OR "two years old"[ti] OR "three years old"[ti] OR "four years old"[ti] OR "five years old"[ti] OR "six years old"[ti] OR "2 years old"[ti] OR "3 years old"[ti] OR "4 years old"[ti] OR "5 years old"[ti] OR "6 years old"[ti]) NOT (animals[Mesh] NOT humans[Mesh]) |
| --- |
| **b.) Web of Science Search Query**  (ALL=Schistosomiasis OR (TI=Schistosom* OR AB=Schistosom*) OR (TI=Bilharzi* OR AB=Bilharzi*) OR (TI="blood fluke*" OR AB="blood fluke*") OR (TI="snail fever*" OR AB="snail fever*") OR (TI="Katayama fever*" OR AB="Katayama fever*")) AND (ALL=Child OR ALL=child OR ALL=children OR ALL=Infant OR ALL=infan* OR ALL=newborn* OR ALL=new-born* OR ALL=baby OR ALL=babies OR ALL=suckling* OR ALL=toddler* OR ALL=childhood OR ALL=schoolchild* OR ALL=childcare OR ALL=child-care OR TI=young OR ALL=youngster* OR ALL=preschool OR ALL=pre-school OR ALL=kid OR ALL=kids OR ALL=boy OR ALL=boys OR ALL=girl* OR ALL=pre-adolescen* OR ALL=schoolage* OR ALL=school-age* OR ALL=schoolboy* OR ALL=schoolgirl* OR ALL=Pediatrics OR ALL=Pediatric* OR ALL=Paediatric* OR (ALL=child NOT AU=child) OR ALL=children* OR ALL=schoolchild* OR ALL="under 5" OR ALL="<5 year*" OR ALL="<=5 year*" OR ALL=infan* OR ALL=pediat* OR ALL=paediat* OR ALL=neonat* OR ALL=toddler* OR ALL=preteen* OR ALL=newborn* OR ALL=postneonat* OR ALL=postnatal* OR ALL=puberty OR ALL=preschool* OR ALL=suckling* OR ALL=juvenile OR ALL="new born*" OR ALL=new-born* OR ALL=neo-nat* OR ALL=neonat* OR ALL=perinat* OR ALL=underag* OR ALL="under age" OR ALL="under aged" OR ALL=youth* OR ALL=kinder* OR ALL=pubescen* OR ALL=prepubescen* OR ALL=prepuberty OR ALL="school age" OR ALL="stratified by age" OR ALL=schoolage OR ALL="school ages" OR ALL=schoolage* OR TI="one year old" OR TI="two year old" OR TI="three year old" OR TI="four year old" OR TI="five year old" OR TI="six year old" OR TI="1 year old" OR TI="2 year old" OR TI="3 year old" OR TI="4 year old" OR TI="5 year old" OR TI="6 year old" OR TI="two years old" OR TI="three years old" OR TI="four years old" OR TI="five years old" OR TI="six years old" OR TI="2 years old" OR TI="3 years old" OR TI="4 years old" OR TI="5 years old" OR TI="6 years old") NOT (ALL=animals NOT ALL=humans) |
| **C.) Embase (Ovid) Search Strategy**  (exp Schistosomiasis/ OR Schistosom*.tw. OR Bilharzi*.tw. OR "blood fluke*".tw. OR "snail fever*".tw. OR "Katayama fever*".tw.) AND (exp Child/ OR child.mp. OR children.mp. OR exp Infant/ OR infan*.mp. OR newborn*.mp. OR new-born*.mp. OR baby.mp. OR babies.mp. OR suckling*.mp. OR toddler*.mp. OR childhood.mp. OR schoolchild*.mp. OR childcare.mp. OR child-care.mp. OR young.ti. OR youngster*.mp. OR pre-school.mp. OR pre-school.mp. OR kid.mp. OR kids.mp. OR boy.mp. OR boys.mp. OR girl*.mp. OR pre-adolescen*.mp. OR schoolage*.mp. OR school-age*.mp. OR schoolboy*.mp. OR schoolgirl*.mp. OR exp Pediatrics/ OR Pediatric*.mp. OR Paediatric*.mp. OR (child.af. NOT child.au.) OR children*.af. OR schoolchild*.af. OR "under 5".mp. OR "<5 years*".af. OR "<=5 year*".af. OR infan*.af. OR pediat*.af. OR paediat*.af. OR neonat*.af. OR toddler*.af. OR preteen*.af. OR newborn*.af. OR postneonat*.af. OR postnatal*.af. OR puberty.af. OR preschool*.af. OR suckling*.af. OR juvenile.af. OR "newborn*".af. OR new-born*.af. OR neo-nat*.af. OR neonat*.af. OR perinat*.af. OR underag*.af. OR "under age".af. OR "under aged".af. OR youth*.af. OR kinder*.af. OR pubescen*.af. OR prepubescen*.af. OR prepuberty.af. OR "school age".af. OR "stratified by age".af. OR schoolage.af. OR "school ages".af. OR schoolage*.af. OR "one year old".ti. OR "two year old".ti. OR "three year old".ti. OR "four year old".ti. OR "five year old".ti. OR "six year old".ti. OR "1 year old".ti. OR "2 year old".ti. OR "3 year old".ti. OR "4 year old".ti. OR "5 year old".ti. OR "6 year old".ti. OR "two years old".ti. OR "three years old".ti. OR "four years old".ti. OR "five years old".ti. OR "six years old".ti. OR "2 years old".ti. OR "3 years old".ti. OR "4 years old".ti. OR "5 years old".ti. OR "6 years old".ti.) NOT (exp animals/ NOT exp humans/) |
| **d.) LILACS search Strategy**  schistosomiasis AND (children OR Preschool child*) |
