# Supplementary material for: Epidemiology of pediatric schistosomiasis in hard-to-reach areas and populations: a scoping review
Source: Infect Dis Poverty. 2023 Apr 17;12:37. doi: 10.1186/s40249-023-01088-x (PMC10108517; doi:10.1186/s40249-023-01088-x)
Supplement: Supplementary file 2 — Additional file 2. Quality assessment report of articles included in the review. [file 40249_2023_1088_MOESM2_ESM.docx]

**Additional file 2:** Quality Assessment Report of Articles Included in the Review

The quality of the included articles was assessed using the Joanna Briggs Institute (JBI) Prevalence Critical Appraisal Tool (1). The tool assessed each article on the following:

1. Sample representative of the target population
2. Study participants recruited in an appropriate way
3. Sample size adequate
4. Study subjects and setting described in detail
5. Data analysis was conducted with enough coverage of the identified sample
6. Validity of methods used to identify the condition of interest
7. Condition measured in a standard, reliable way for all participants
8. Appropriate statistical analysis
9. Adequacy of response rate; were low response rates managed?
10. Sub-population of interest identified using objective criteria

| Author(s) and Year | Was the sample representative of the target population? | Were study participants recruited in an appropriate way? | Was the sample size adequate? | Were the study subjects and setting described in detail? | Was data analysis conducted with sufficient coverage of the identified sample? | Were valid methods used for the identification of the condition? | Was the condition measured in a standard, reliable way for all participants? | Was there appropriate statistical analysis? | Was the response rate adequate, and if not, was the low response rate managed appropriately? | Were target subpopulations identified using objective criteria? | Quality score |
| --- | --- | --- | --- | --- | --- | --- | --- | --- | --- | --- | --- |
| Sassa et al., 2020 | Yes | Yes | Yes | Yes | Yes | Yes | Yes | Yes | NA | Yes | 9 |
| N'Diaye et al., 2016 | No | Yes | No | Yes | Yes | Yes | Yes | Yes | NA | Yes | 7 |
| Mafiana, Ekpo and Ojo 2003 | Yes | Yes | Yes | Yes | Yes | Yes | Yes | Yes | NA | Yes | 9 |
| Kabatereine et al., 2014 | Yes | Yes | Not specified | Yes | Yes | Yes | Yes | Yes | NA | Yes | 8 |
| Hodges et al., 2012 | Yes | Yes | Yes | Yes | Yes | Yes | Yes | Yes | NA | Yes | 9 |
| Davis et al., 2015 | Yes | No | Yes | Yes | Yes | Yes | Yes | Yes | NA | Yes | 8 |
| Akosah-Brempong et al., 2021 | Yes | Yes | No | Yes | Yes | Yes | Yes | Yes | NA | Yes | 8 |
| Sheehy et al., 2021 | Yes | No | No | Yes | Yes | Yes | Yes | Yes | NA | Yes | 7 |
| Ruganuza et al., 2015 | Yes | Yes | Yes | Yes | Yes | Yes | Yes | Yes | NA | Yes | 9 |
| Green et al., 2011 | Yes | Not specified | Yes | Yes | Yes | Yes | Yes | Yes | NA | Yes | 8 |
| Nalugwa et al., 2017 | Yes | Yes | Yes | Yes | Yes | Yes | Yes | Yes | NA | Yes | 9 |
| Nalugwa et al., 2015 | Yes | Yes | Yes | Yes | Yes | Yes | Yes | Yes | NA | Yes | 9 |
| Mueller et al., 2019 | Yes | Yes | Yes | Yes | Yes | Yes | Yes | Yes | NA | Yes | 9 |

**Reference**

1. Munn Z, Moola S, Riitano D, Lisy K. The development of a critical appraisal tool for use in systematic reviews addressing questions of prevalence. Int J Health Policy Manag. 2014;3(3):123-8.
